# T/T homozygosity of the *tenascin-C* gene polymorphism rs2104772 negatively influences exercise-induced angiogenesis

**DOI:** 10.1371/journal.pone.0174864

**Published:** 2017-04-06

**Authors:** Paola Valdivieso, Marco Toigo, Hans Hoppeler, Martin Flück

**Affiliations:** 1 Laboratory for Muscle Plasticity, Department of Orthopedics, University of Zurich, Balgrist Campus, Zurich, Switzerland; 2 Institute of Anatomy, University of Berne, Berne, Switzerland; University of Birmingham, UNITED KINGDOM

## Abstract

**Background:**

Mechanical stress, including blood pressure related factors, up-regulate expression of the pro-angiogenic extracellular matrix protein tenascin-C in skeletal muscle. We hypothesized that increased capillarization of skeletal muscle with the repeated augmentation in perfusion during endurance training is associated with blood vessel-related expression of tenascin-C and would be affected by the single-nucleotide polymorphism (SNP) rs2104772, which characterizes the non-synonymous exchange of thymidine (T)-to-adenosine (A) in the amino acid codon 1677 of *tenascin-C*.

**Methods:**

Sixty-one healthy, untrained, male white participants of Swiss descent performed thirty 30-min bouts of endurance exercise on consecutive weekdays using a cycling ergometer. Genotype and training interactions were called significant at Bonferroni-corrected p-value of 5% (repeated measures ANOVA).

**Results:**

Endurance training increased capillary-to-fiber-ratio (+11%), capillary density (+7%), and mitochondrial volume density (+30%) in *m*. *vastus lateralis*. Tenascin-C protein expression in this muscle was confined to arterioles and venules (80% of cases) and increased after training in A-allele carriers. Prior to training, volume densities of subsarcolemmal and myofibrillar mitochondria in *m*. *vastus lateralis* muscle were 49% and 18%, respectively, higher in A/A homozygotes relative to T-nucleotide carriers (A/T and T/T). Training specifically increased capillary-to-fiber ratio in A-nucleotide carriers but not in T/T homozygotes. Genotype specific regulation of angiogenesis was reflected by the expression response of 8 angiogenesis-associated transcripts after exercise, and confirmed by training-induced alterations of the shear stress related factors, vimentin and VEGF A.

**Conclusion:**

Our findings provide evidence for a negative influence of T/T homozygosity in rs2104772 on capillary remodeling with endurance exercise.

## Introduction

Tenascin-C is an anti-adhesive extracellular matrix glycoprotein, with a spatial-temporally restricted expression in tissues that undergo active remodeling and angiogenesis during development (reviewed in [1, 2]) and in consequence of cancer, wound healing and hypertension [3–7]. Tenascin-C thereby acts as permissive factor for morphogenic processes by relieving the decorated cells from the mechanical constraints of contact inhibition, which suppress protein synthesis and proliferation [8].

In the healthy adult animal, *tenascin-C* expression is mainly detectable in musculoskeletal tissues [9], sensory and motor nerves [7, 10], and blood vessels [11, 12]. Especially high tenascin-C expression is found at branching sites of blood vessels [7, 13] where blood flow is disturbed [14]. Blood-flow related expression of tenascin-C is supported by the sharp up-regulation of the *tenascin-C* transcript in smooth muscle and endothelial cells in response to shear stress [15, 16] and increased *tenascin-C* transcript expression in knee extensor muscle after cycling-type endurance exercise under inhibition of angiotensin-mediated vasoconstriction [17] which augments tissue perfusion [18]. These observations implicate tenascin-C in the regulation of physiological angiogenesis subsequent to repeated increases in blood flow with endurance training [19, 20]. This adaptation typically becomes manifest after a few weeks of training through an increase in capillary volume density and capillary-to-fiber ratio in exercised muscle [21]. The underlying biological process is reflected by altered muscle transcript expression during the first 24 hours of recovery from aerobic exercise for factors being associated with angiogenesis-related remodeling of the extracellular matrix [22, 23].

The regulation of *tenascin-C* expression in skeletal muscle has been manly studied in situations, which damage muscle fibers. For instance, massively enhanced tenascin-C expression has been documented in response with dystrophic disease [24] and in response to eccentric types of contraction, which strain muscle fibers beyond resting length during force production, [25–27]. The thereby elevated tenascin-C content is associated with the endomysial connective tissue layer of the consequently injured muscle fibers [28, 29]. Using transgenic mice we have shown that in this situation tenascin-C develops pleiotropic actions on myogenesis, wound healing and angiogenesis which allow repair of damaged muscle [30].

Rs2104772 is a SNP within the *tenascin-C* gene that is associated with a higher incidence for pathological remodeling of airways in asthma and Achilles tendon rupture [31, 32], which are both associated with aberrant vascular remodeling [33, 34]. The SNP describes a thymidine (T)-to-adenosine (A) exchange at nucleotide position 44513 in the tenascin-C gene that instructs the substitution of leucine by isoleucine at amino acid position 1677 [31]. A number of SNPs are now being identified to influence phenotypic variation in adaptation of physiological parameters to physical training [35], including the implicated plasticity of muscle tissue [36, 37]. In this regard rs2104772 is a candidate SNP affecting the adaptive responses in skeletal muscle to endurance training, such as the remodeling of capillaries and associated subcellular compartments of skeletal muscle, and the related system parameter of VO2max. The study of SNP related traits and underlying gene expression (genetical genomics) has been proposed as powerful approach to expose mechanistically important gene regulation [38].

Here we used a genetic approach with the aim of confirming the suggested role of tenascin-C in endurance training-induced remodeling of capillaries in human skeletal muscle. We first asked whether tenascin-C expression in *m*. *vastus lateralis* would increase in association with the vasculature after cycling type endurance training. Subsequently we investigated whether SNP rs2104772 would affect alterations in muscle capillarization and the dependent mitochondrial organelle [39] with endurance training and would produce alterations in angiogenesis-associated transcript expression during recovery from exercise. Because of the documented association of the A/A genotype with asthma [31], which is associated with an increased capillarization (reviewed in [33]), we hypothesized that the A/A genotype of SNP rs2104772 demonstrates an accentuated increase in muscle capillarization after training.

## Material and methods

### Design

This study was performed with 61 healthy, recreationally active young (mean age of 30 years), male white participants of Swiss descent from the Cantons of Berne and Fribourg without prior experience in endurance training (Table 1). The study participants were asked to refrain from strenuous physical activity for 2–4 weeks before the start of the study and endurance training program. Basic anthropometry and aerobic performance were assessed by a single bout of cycle ergometer exercise 3 weeks before and 3–4 d after a 6-weeks endurance-training program. On this occasion muscle biopsies were collected with a Bergström needle from the mid portion of *vastus lateralis* muscle for all subjects. In 12 participants additional *vastus lateralis* muscle biopsies were collected from the mid portion with fine needles (14 gauge single use; Medilink, Pregassona, Switzerland) 1, 8 and 24 h after the first bout of exercise, alternately from the left and right leg. For each biopsy a fresh incision was made at a distance of at least 1.5 cm from any previous biopsy of the same leg. In the subsequent week, the participants commenced the training. Bergström needle biopsies were used for the characterization of tissue composition using morphometry, tenascin-C and vimentin protein expression, and for the genotyping of SNP rs2104772 within the *tenascin-C* gene. Bergström needle biopsies from the pre-exercise time point and fine needle biopsies were used to measure transcript expression in the subset of subjects for which sufficient biopsy material was available.

**Table 1:** Effect of rs2104772 on muscle-related variables prior to endurance training.

*Parameter*	*ALL (n = 61)*	*A/A (n = 12)*	*A/T (n = 38)*	*T/T (n = 11)*	*p-value*		*T*
					*ALL*	*A*	
Age [y]	29.5 ± 9.3	31.3 ± 8.3	27.9 ± 8.3	33.7 ± 12.8	0.199	0.245	0.877
Height [cm]	178.8 ± 6.6	177.5 ± 8.0	179.6 ± 6.5	177.1 ± 5.3	0.481	0.574	0.718
Body mass [kg]	76.5 ± 12.7	75.1 ± 11.8	76.4 ± 13.3	78.2 ± 12.9	0.868	0.611	0.640
BMI [kg m^–2^]	23.9 ± 3.3	23.7 ± 2.2	23.6 ± 3.3	25.0 ± 4.3	0.536	0.295	0.631
Pmax [W]	303.9 ± 38.8	285.4 ± 52.7	308.0 ± 29.9	303.7 ± 57.9	0.685	0.712	0.307
VO2max [ml O_2_ min^–1^ kg^–1^]	45.5 ± 8.9	45.1 ± 9.5	46.3 ± 8.0	43.1 ± 12.1	0.639	0.448	0.893
Capillary-to-fiber ratio	1.9 ± 0.6	1.8 ± 0.6	1.9 ± 0.6	2.0 ± 0.8	0.726	0.515	0.434
Capillary density [mm^–2^]	496.2 ± 92.5	513.6 ± 100.6	494.0 ± 98.0	484.6 ± 64.8	0.741	0.553	0.443
Muscle fiber area [μm2]	3592.7 ± 1158.7 1158.7	3177.1 ± 453.6	3633.5 ± 975.5	3908.8 ± 1986.5 1986.5	0.305	0.210	0.131
Myofibrils [%]	80.4 ± 3.1	79.1 ± 3.8	80.7 ± 2.9	80.7 ± 3.1	0.274	0.434	0.122
**Total mitochondria [%]**	5.2 ± 1.3	6.0 ± 1.4	5.0 ± 1.1	5.3 ± 1.5	0.054	0.692	**0.047**
If-mitochondria [%]	4.3 ± 0.9	4.7 ± 0.9	4.1 ± 0.8	4.3 ± 1.1	0.132	0.802	0.108
**Ss-mitochondria [%]**	0.9 ± 0.5	1.3 ± 0.7	0.9 ± 0.4	0.8 ± 0.6	**0.039**	0.151	**0.013**
IMCL [%]	0.6 ± 0.3	0.6 ± 0.4	0.6 ± 0.3	0.4 ± 0.1	0.124	0.056	0.412
Residual organelles [%]	13.8 ± 2.3	14.4 ± 3.0	13.7 ± 2.2	13.6 ± 2.0	0.862	0.558	0.352

Data refer to mean ± standard deviation (SD), sample number (n), and *p*-values for the effect of the rs2104772-genotype and the presence of adenosine (A) or thymidine (T) on anthropometric and muscle related parameters before endurance training. Data were assessed with an ANOVA with post hoc test of Fisher. BMI, body mass index; if-mitochondria, intramyofibrillar mitochondria; IMCL, intramyocellular lipids; Pmax, maximal aerobic power output; ss-mitochondria, subsarcolemmal mitochondria; VO2max, maximal oxygen uptake; % refers to volume densities per fiber volume. Differences which passed a Bonferroni post hoc test at a *p*-value of < 0.05, and the name of the corresponding parameter, appear in bold, underlined font.

### Ethics

The ethics committee of the Canton of Berne (Berne, Switzerland) approved the study protocol. All investigations were performed in accordance with the ethical standards of the 1964 Declaration of Helsinki. Written informed consent was obtained from every participant.

### Endurance test

Peak oxygen uptake and the maximal cycling power (Pmax) was assessed with ergospirometry. Two weeks before the first bout of exercise, subjects were familiarized with the test equipment. Exercise tests were conducted using a cycle ergometer (Ergoline 800S; Ergoline, Bitz, Germany). Expired air was analyzed with breath-by-breath measurements (Oxycon alpha; Jäger, Würzburg, Germany) and heart rate was monitored using an Accurex Plus chest belt (Polar Electro Finland, Kempele, Finland). Starting at 40 W, and a cadence of 70–80 rpm, power was increased by 30 W every 2 min until the participants could no longer maintain a cadence of over 60 rpm. At the end of each 2-min increment in power, a capillary sample was taken from the ear lobe and analyzed for blood lactate essentially as described [21]. Peak oxygen uptake was determined as the highest (mean) value of oxygen uptake after the increase in oxygen uptake levelled off and blood lactate reached at least 10 mM.

### Endurance training

Participants trained 5 times per week on consecutive days (Monday through Friday) for 6 weeks. Each training session consisted of 30 min cycle ergometer exercise at a heart rate corresponding to 65% of Pmax. Training intensity was monitored based on heart rate (Accurex Plus, Polar Electro Finland, Kempele, Finland) and increased as necessary to maintain a constant individual training intensity at approximately 90% of maximal heart rate. Cycling power was increased as necessary to maintain a constant training heart rate corresponding to 83 ± 1% of the individual’s maximal heart rate in the first training week, and to 90 ± 2% in the sixth training week.

### Muscle biopsies

Before and after training, muscle tissue was collected from the mid-portion of the *vastus lateralis* muscle using a modified Bergström biopsy device with local anesthesia administered via subcutaneous injection of 3–5 ml of 2% lidocaine (Rapidocain Lidocaini HCl 10mg/ml, Sintetica; Mendrisio, Switzerland) as previously described [21]. Bergström needle biopsies were trimmed in two parts; the major part was immediately frozen in liquid nitrogen cooled isopentane, and then stored in the latter until required for analyses. The other part was fixed in glutaraldehyde and processed for electron microscopy and morphometry (see the ‘morphometry’ paragraph). Fine needle biopsies were immediately frozen in isopentane cooled liquid nitrogen and stored in liquid nitrogen.

### Genotyping

The targeted SNP rs2104772 was analyzed using high-resolution melt PCR (HRM-PCR). Genomic DNA was extracted from ~1 mm^3^ cryosections of the muscle biopsy using the DNeasy Blood and Tissue Kit (Cat. No 69504, Qiagen, Basel, Switzerland) following the manufacturer’s protocol, essentially as previously described [36]. DNA concentration and purity were measured using a NanoDrop USV-99 AGTGene (Labgene Scientific, Châtel-St-Denis, Switzerland). Absorbance measurements at 260 nm and 280 nm indicated that the final DNA concentration ranged from 10 to 50 ng/μL and that the DNA was of high purity. DNA samples were diluted to a final concentration of 5 ng/μL and stored at −20°C until analysis. Genotyping was performed with HRM-PCR combined with sequencing. Available online tools (i.e., Primers-BLAST: http://www.ncbi.nlm.nih.gov/tools/primer-blast/; and Primer 3 output: http://primer3.ut.ee) were used to design oligonucleotide primers to target the *tenascin-C* polymorphism (rsNCBI: rs2104772; SNP-44513-AT). The designed primers (5′-CAAAAAGCAGTCTGAGCCAC-3′ and 5′-TTCAGTAGCCTCTCTGAGAC-3′) amplified a 85-bp fragment containing the SNP rs2104772.

Specificity of the PCR reaction was validated in experiments with negative and positive controls and by confirming the predicted PCR product size via agarose gel electrophoresis. This was followed by DNA band isolation and subsequent DNA sequencing by Microsynth (Balgach, Switzerland). The negative control was a non-template control reaction (NTC) with DNAse-free water and positive controls comprised genotyped DNA samples, including samples for each genotypic variant of SNP rs2104772 (i.e., A/T, A/A, and T/T). The positive controls also served as internal references to generate a melting profile for screening unknown samples.

HRM-PCR reactions were run in duplicate. The reaction mix included 10 ng DNA template, 1× KAPA HRM FAST Master Mix (KAPA BIOSYSTEMS, Labgene Scientific, Châtel-St-Denis, Switzerland), 2.5 mmol MgCl_2_, 0.2 μmol of each primer, and water up to a final volume of 10 μL. Amplification and melt curve analysis was then performed using an EcoTM Thermal and Optical system (Illumina; Labgene Scientific, Châtel-St-Denis, Switzerland). The reaction conditions were as follows: 3 min enzyme activation at 95°C; followed by 35 amplification cycles of 5 s denaturation at 95°C and 30 s annealing/extension at 60°C; and a final melting cycle of heating to 95°C, cooling to 55°C, and ending at 95°C. For each cycle, the fluorescent signal from the EvaGreen-dye contained in the 1× KAPA HRM FAST Master Mix was analyzed using EcoStudy software (Illumina, Labgene Scientific, Labgene Scientific, Châtel-St-Denis, Switzerland). Raw data of the melting curves were normalized versus the A/A genotype, the samples clustered, and displayed as raw values and derivative normalized melting curves and peaks.

### Transcript profiling

Total RNA was isolated from 10 mm^3^ biopsy material from the pre, 1, 8 and 24 h post exercise samples using an RNeasy Mini Kit (Qiagen, cat N° 74104, Basel, Switzerland) and subjected to microarray analysis with a validated, custom-made low-density Atlas^®^cDNA array (BD Biosciences, Allschwil, Switzerland; GPL 1935 in GEO, http://www.ncbi.nlm.nih.gov/geo). The array covered 231 gene transcripts from major gene ontologies related to energy and protein metabolism, muscle structure, cell regulation and angiogenesis. Gene ontologies were curated based on information available through public databases (http://www.uniprot.org/uniprot; www.expasy.org; http://www.ncbi.nlm.nih.gov/pubmed). Data sets were deposited under provisional accession codes GSE 13623 and GSE 2479, respectively, at GEO.

### Detection of protein expression

Total protein homogenate was prepared from biopsy material and subjected to SDS-PAGE and immunoblotting for tenascin-C, vimentin or VEGF A essentially as previously described [30]. Briefly, 10 mm^3^ of muscle tissue was cross-sectioned to 25 μm using a cryostat and mixed with ice-cold RIPA buffer that included 10 mM Tris-HCl (pH 7.5), 150 mM NaCl, 1 mM EDTA, 1% NP-40, 2% Triton X100, 2 mM EDTA, 2 mM EGTA, one PhosStop tablet, and one tablet of Complete-mini EDTA-Free reagent (Roche Diagnostics, Mannheim, Germany). This mixture was homogenized using a Polytron^®^PT 1200E hand-held homogenizer (Kinematica, Lucerne, Switzerland). The amount of protein in the total homogenate was determined using the BCA method (Pierce, Rockford, IL, USA) against bovine serum albumin (BSA) as a standard. The total homogenate was adjusted to a concentration of 2 μg per μL using Laemmli buffer (Biorad Laboratories, Cressier, Switzerland) and 2% mercaptoethanol, and the mixture was heated at 95°C for 5 min.

Next, 20 μg total protein was separated on hand-made 7.5% polyacrylamide gels in a Mini-Protean III electrophoretic system (BioRad Laboratories, Cressier, Switzerland). Samples of pre- and post-training pairs were loaded separately in adjacent lanes, with four sample pairs loaded per gel. Proteins were subsequently transferred onto nitrocellulose membrane (Protean, GE Healthcare Europe, Glattbrugg, Switzerland), the blotting efficiency was visualized by Ponceau S staining of the actin band near 45 kDa. Immunodetection was performed using the monoclonal tenascin-C antibody B28.13 (a gift from Prof. Matthias Chiquet; 1:50 dilution), monoclonal vimentin antibody MAB1681 (MERCK Millipore, Schaffhausen, Switzerland, 1:1000 dilution), or monoclonal VEGF A antibody 26503 (Abcam, Cambridge, UK, 1: 500 dilution) and anti-mouse secondary horseradish peroxidase-conjugated antibody (1:5000 dilution of A-2304 or 1:20’000 dilution of A-9917 from Sigma, Buchs Switzerland). The bands corresponding to the tagged proteins were detected using chemoluminescence (Femto kit; Pierce, Fisher Scientific, Wohlen, Switzerland) and recorded using a Chemidoc system with Quantity One software (Bio-Rad, Hercules, CA, USA). Tenascin-C protein content was estimated for the monomer of the 230-kDa isoform [26]. Vimentin protein content was estimated combined for the small and large isoform [40]. VEGF A content was estimated based on the VEGF A dimer (VEGF A_2_) as previously established [24, 41]. Band signal intensity was estimated as pixel intensity per pixel area using the “volume rectangular tool” and was corrected versus the background of a band of equal height and size (area) from an empty sample lane. Background-corrected data were related to actin, and then normalized to the mean values of the pre-training sample from the same gel. Therefore, the final values reflect the relative content per actin.

### Tenascin-C protein localization

Cryosections were prepared from biopsies of *vastus lateralis* muscle, and subjected to immunological staining for tenascin-C using affinity purified rabbit antibody MA3 (gift of Prof. M. Chiquet) or monoclonal antibody B28.13 (gift of Prof. R. Chiquet-Ehrismann), followed by incubation with horse radish peroxidase-conjugated secondary antibody anti-rabbit antibody from goat (#55676, MP Biomedicals, Zurich, Switzerland) or anti-mouse antibody from goat (A-9917, Sigma, Buchs Switzerland) and counterstaining of nuclei with hematoxylin. This reaction was carried out at room temperature essentially as described but with the modification that the first antibody was incubated over night at a 1:100 dilution at 4–8°C [28]. Control reactions were carried out with incubations of a 1:500 dilution of pre-immune serum from a rabbit. Subsequently, the entire area of the stained cross-section was visualized and digitally recorded using an Olympus IX50 microscope and digital camera DP72, which was operated with the CellSens Dimension software (Olympus, Volketswil, Switzerland). The assembled image was printed in color and tenascin-C positive structures were manually counted. Biopsy samples from the pre- and 24 h post exercise time point before, and after training, were analyzed. On average 122 muscle fibers were assessed per biopsy cross-section. Tenascin-C and CD31 co-expression was detected using immunofluorescence essentially as described after co-incubation of the slides with a 1:10 dilution of monoclonal mouse antibody B28.13 and a 1:50 dilution of rabbit antibody ab28364 (CD31, Abcam, Cambridge, UK) overnight at 4–8°C followed by washes in PBS and incubation with respective secondary antibodies (Alexa Fluor 488 anti-mouse antibody (A11017, Life Technologies) and Alexa Fluor 555 anti-rabbit antibodies from goat (A21428, Life Technologies) for 1 hour at room temperature. Nuclei were stained with Hoechst 33342 (Thermo Fisher Scientific, Reinach, Switzerland). Signals were visualized using standard fluorescent modules (U-MWU, U-MWG, U-MWBV) on the Olympus IX50 microscope with the help of a UV lamp, recorded and assembled using the Cell Sens Dimension software (Olympus, Volketswil, Schweiz).

### Morphometry

**Fig 1:**
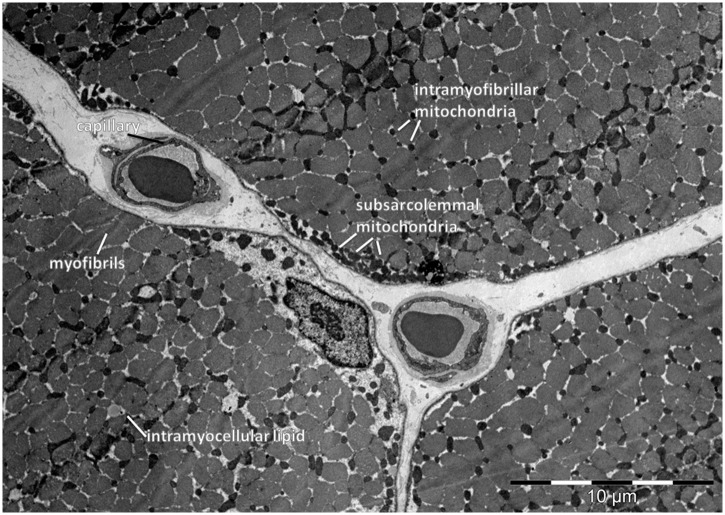
Assessed myocellular parameters. Electron micrograph indicating the assessed ultrastructural parameters in vastus lateralis muscle of a subject before endurance training.

Glutaraldehyde-fixed samples were embedded in Epon resin (Sigma Aldrich Chemie GmbH, Buchs, Switzerland) and processed for electron transmission microscopy and morphometric analyses as described elsewhere [21, 42]. Volume densities of myofibrils, intramyofibrillar and subsarcolemmal mitochondria and intramyocellular lipids were estimated at a final magnification of ×24,000 on electron micrographs by performing point counting with a B36 grid of 144 test points. Fig 1 illustrates the assessed parameters. Capillary measurements and muscle fiber typing was performed on ATPase stained cross sections of frozen muscle biopsies as described elsewhere [42]. The morphometric data was submitted to the Open Science Framework (study MORPHOMEXX; https://osf.io/nhaxr/).

### Statistics

All statistical analyses were performed using SPSS (vs.22 IBM, Zurich, Switzerland). Differences between rs2104772 genotypes (i.e. A/A, A/T, or T/T) prior to training were assessed with a one-way ANOVA. Changes between the pre- and post-training values were analyzed with a repeated ANOVA for the factor “rs2104772 genotype” and the repeated factor “training” (yes or no). Effects were localized using a Bonferroni post-hoc test. Compliance with Hardy-Weinberg equilibrium was assessed using an online calculator, i.e. http://www.had2know.com/academics/hardy-weinberg-equilibrium-calculator-2-alleles.html. Genotype dependence of vimentin protein, respectively, was analyzed for the combined data for the small and large isoform. Relationships were calculated based on Spearman’s rank correlations and called significant at p < 0.05. The significance level of differences in transcript expression was assessed based on normalized values of transcript expression using permutations of T-tests with the Significance Analysis of Microarrays test (SAM) running as an applet in MS-Excel [43] based on the specific design of the comparison. This included a ‘one class time course analysis’ to identify transcripts which expression altered during the first 24 hours of recovery from exercise. Genotype effects were identified by a subsequent ‘unpaired one class analysis’ on fold changes in expression 24 h post exercise for the regulated set of transcript. Only those transcripts were accepted which demonstrated opposite regulation between genotypes (i.e. up-regulated in the A/A genotype down-regulated in the T/T genotype, or inverse). A false discovery rate of 1% was deemed appropriate. Heat maps were produced through the use of Cluster and Treeview software essentially as described [44]. Statistical power was assessed using the freely available G*Power software (version 3.1.9.2; http://www.gpower.hhu.de).

## Results

### Effect of endurance training

Table 1 presents the anthropometric and muscle related data for the 61 participants. Endurance training on the cycle ergometer improved V̇O_2_max (+9%) and local components of aerobic fitness in the *vastus lateralis* muscle, including the capillary-to-fiber ratio (+11%), capillary density (+7%), and mitochondrial volume density (+30%; Table 2).

**Table 2:** Muscle-related adjustments of subjects with endurance training, and the interaction effect of the polymorphism rs2140772.

*Parameter*	*ALL (n = 61)*	*A/A (n = 12)*	*A/T (n = 38)*	*T/T (n = 11)*	*p-value*
Pmax [W]	38.71 ± 17.52 *	41.40 ± 25.21 *	39.57 ± 14.52 * 14.52 *	33.17 ± 23.29 * 23.29 *	0.431
VO2max [ml O_2_ min^–1^ kg^–1^]	3.87 ± 2.8*	4.16 ± 3.24*	4.06 ± 2.82 *	2.67 ± 2.10 *	0.277
**Capillary-to-fiber ratio**	**0.23 ± 0.53** *	**0.45 ± 0.82**	**0.24 ± 0.38** *	**-0.25 ± 0.08**	**0.004**
Capillary density [mm^–2^]	27.4 ± 94.7 *	44.5 ± 95.5	34.4 ± 99.0 *	-12.4 ± 74.2	0.118
Muscle fiber area [μm2]	300.5 ± 1438.3 1438.3	204.8 ± 890.0 890.0	472.1 ± 1441.1 1441.1	-165.0 ± 1837.5 1837.5	0.545
Myofibrils [%]	-3.33 ± 3.73*	-3.24 ± 4.95	-3.81 ± 3.55 *	-1.88 ± 2.71	0.265
Total mitochondria [%]	1.42 ± 1.30 *	1.52 ± 1.86 *	1.54 ± 1.06 *	0.89 ± 1.40	0.172
If-mitochondria [%]	0.96 ± 0.89 *	0.80 ± 1.10	1.07 ± 0.73 *	0.76 ± 1.16	0.196
Ss-mitochondria [%]	0.49 ± 0.78*	0.72 ± 1.12	0.48 ± 0.64*	0.29 ± 0.87	0.375
IMCL [%]	0.15 ± 0.38 *	0.10 ± 0.45	0.15 ± 0. 93*	0.22 ± 0.30*	0.310
Residual organelles [%]	1.76 ± 2.88*	1.62 ± 3.29	2.11 ± 2.90 *	0.76 ± 2.35	0.310

Data refer to mean ± standard deviation (SD), biological replicas (n), and *p*-values for the absolute changes (i.e. delta post vs. pre-training) of muscle related parameters between rs2104772 genotypes.

* indicates a significant difference post vs. pre training.

Differences, which passed a false discovery rate adjusted *p*-value of 0.05, and the corresponding parameter, are underlined. For abbreviations and further detail see Table 1.

### SNP rs2104772 and muscle composition at baseline

**Fig 2:**
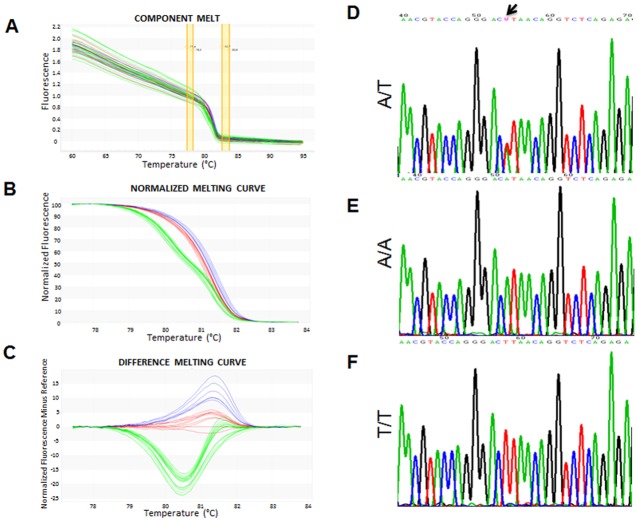
Analysis of SNP rs2104772 by High-Resolution Melt analysis (HRM). *A-C)* Line graph showing the detected or derived fluorescence intensity for the analyzed SNP (i.e. melting curves). The displayed examples included measurements for A/A homozygotes (red line, n = 5)) and T/T homozygotes (blue line, n = 3) relative to the heterozygote A/T (green line, n = 12). Every sample was analyzed in duplicate. A) Raw data of the pre-melt, melt, and post-melt regions. B) Normalized data derived from the raw data plots. C) Melting curves derived after normalization versus the A/A genotype. *D-F)* Sequence analysis of the three identified genotypes in chromatograms presenting the forward sequence. Arrows link the single-nucleotide polymorphism (SNP) position 44513. The presence of 'W' in A/T genotype denotes heterozygosis for the SNP where a double-peak is present at position 44513 for both sequenced alleles (arrow, nucleotides A and T simultaneously).

We assessed the influence of SNP rs2104772 on cellular parameters being related to capillary supply lines in the exercised muscle (Fig 1; Table 2). Fig 2 presents an example of the characterization of the rs2104772 genotype based on the presence, or absence, of thymidine or adenosine at nucleotide position 44513 within the *tenascin-C* gene. Statistical analysis revealed that we could not reject the assumption that the study population was in Hardy-Weinberg equilibrium (*P* = 0.059, n = 61).

Before training, the volume densities of subsarcolemmal and myofibrillar mitochondria in *vastus lateralis* were 50% and 13% higher in A/A homozygotes than T-nucleotide carriers (A/T or T/T; Table 1).

### SNP rs2104772 affects training-induced gains in muscle capillarization

**Fig 3:**
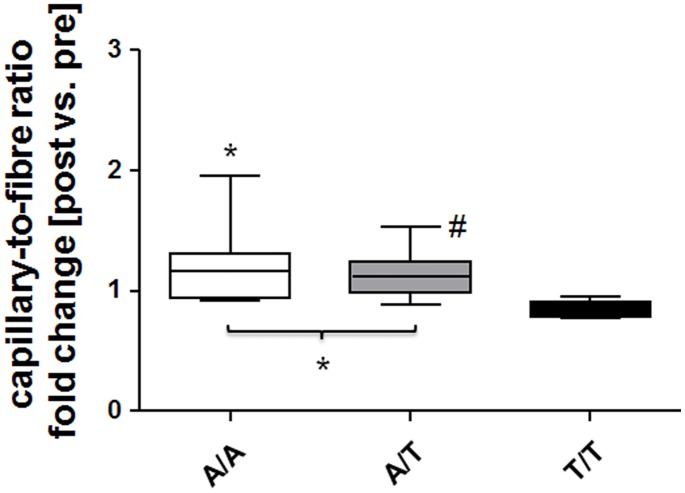
rs140772 affects changes in muscle capillarization with endurance training. Box whisker plot visualizes the medians ± standard errors (central lines and boxes, respectively) and minima/maxima (whiskers) of the fold changes in capillary-to-fiber ratio post- vs. pre-training for the three rs2104772 genotypes. A/A (*n* = 12), A/T (*n* = 38), and T/T (*n* = 11). *, *P* < 0.05 vs. T/T, ANOVA with Fisher’s post-hoc test. #, *P* < 0.05 for post vs. pre for the indicated comparison.

Adjustments in capillary-to-fiber ratio with endurance training demonstrated an interaction effect between endurance training x polymorphism rs140772 (Table 2). Fig 3 resolves the post hoc differences for the training-induced changes for the three respective genotypes of polymorphism rs140772, showing that capillary-to-fiber ratio was increased after the training in subjects with an ‘A-allele’ (i.e., the A/T and A/A genotype) but decreased in comparison by 15% in the study participants with T/T genotype (Fig 3).

### Tenascin-C expression before and after endurance training

**Fig 4:**
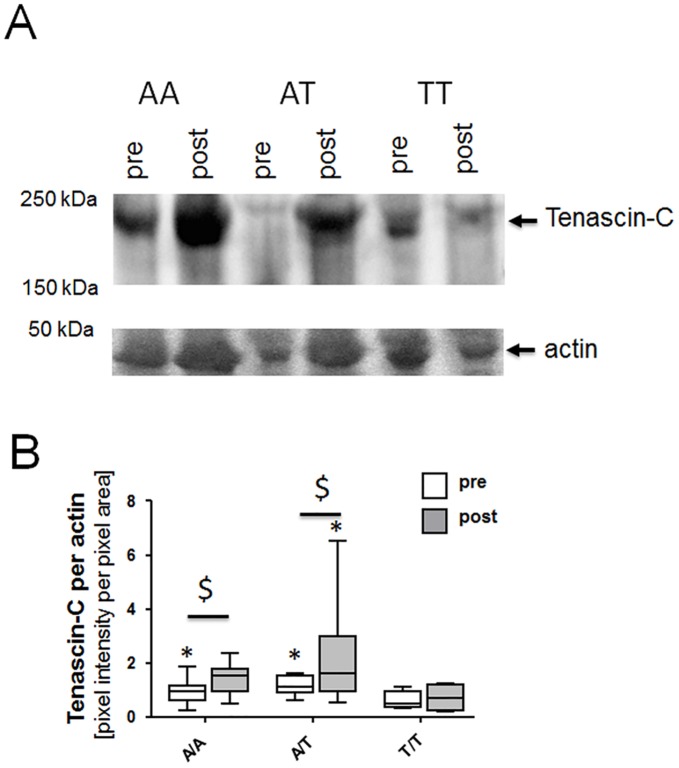
Tenascin-C protein in vastus lateralis muscle with endurance training. (*A*) Immunoblot (Top part of panel) showing the detection of tenascin-C protein in the three rs2104772 genotypes before and after endurance training. The position corresponding to the large tenascin-C isoform at 230 kDa is indicated. The bottom part of the panel shows the corresponding actin band (loading control) on the Ponceau-S-stained membrane before immunoblotting. For image assembly see S2, S3 and S4 Figs. (*B*) Box whisker plot visualizing medians ± standard errors (central lines and boxes, respectively) and minima/maxima (whiskers) of actin-related Tenascin- content pre and post training in the respective genotypes. In total biopsies from 18 subjects were assessed: A/A (*n* = 4), A/T (*n* = 10), and T/T (*n* = 4). *, p < 0.05 vs. T/T pre; $, p < 0.05 vs. pre. ANOVA with Fisher’s post-hoc test.

The 230-kDa isoform of Tenascin-C was abundantly detected in *vastus lateralis* ([26]; Fig 4). Before training, a genotype effect was evident in *vastus lateralis* muscle for the relative content of tenascin-C protein vs. actin (Fig 4). Tenascin-C content was 37% lower in individuals with the T/T genotype compared to those with A/T genotype (p = 0.018). Correspondingly, the transcript level of *tenascin-C* was 41% and 40% lower in the T/T genotype than the A/T and A/A genotype, respectively.

**Fig 5:**
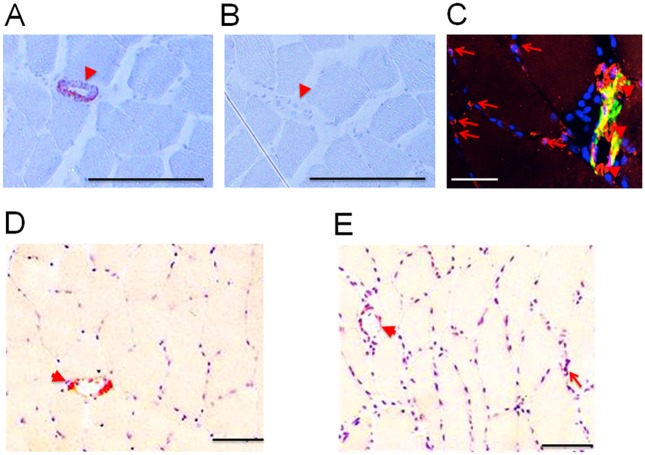
Tenascin-C protein expression in vastus lateralis muscle. *A*,*B)* Controls showing Tenascin-C specific straining (orange) of a large blood vessel (arteriole) in a muscle cross-section of an untrained participant after incubation with antibody MA3 (A) compared to incubation with a non-specific pre-immune serum (B). C) Tenascin-C (green, antibody B28.13) and CD31 (red) immuno-fluorescent staining, as well as co-localized tenascin-C and CD31 staining (yellow) in a section from a trained subject. A large blood vessel (most likely a venule) is identified based on CD31-staining and its thickened vessel wall. D,E) Tenascin-C staining (antibody MA3) in *vastus lateralis* muscle of a same participant before (D) and after (E) endurance training. Nuclei are stained in blue. Arrows and arrowheads point to tenascin-C staining in capillary structures and larger blood vessels (arterioles and venules). Bar = 50 μm.

Endurance training increased the content of tenascin-C protein in carriers of the A-allele (A/A, + 138%; A/T: + 77%). Tenascin-C content remained unchanged in T/T homozygotes (p = 0.54). Tenascin-C staining was associated with capillary structures and CD31-positive arterioles and venules, and the interstitium of muscle fibers (Fig 5). Eighty percent of the staining was confined to arterioles and venules.

### Genotype effect on angiogenesis-associated transcript expression

**Fig 6:**
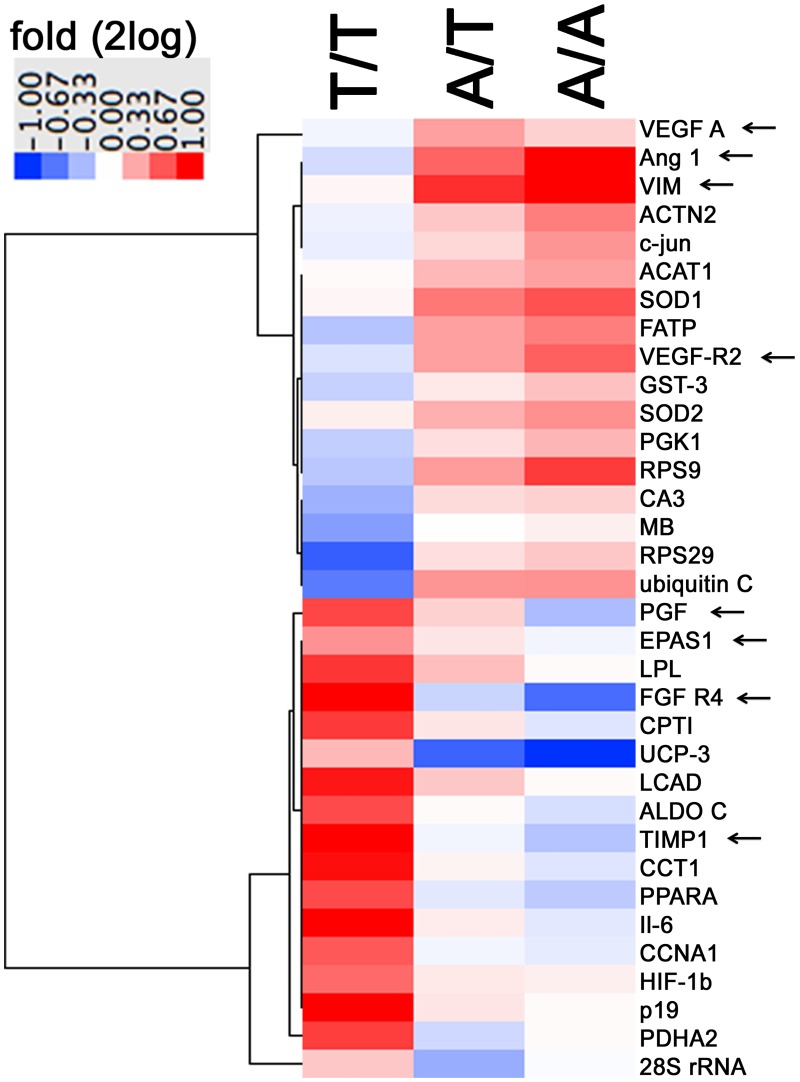
rs140772-dependent alterations in transcript expression. Heat map visualizing fold changes in the expression of the 34 gene transcripts, which demonstrated genotype specific alterations 24 h post exercise. The identified transcripts demonstrated level alterations during the course of the first 24 h after exercise and showed genotype specific differences in fold changes 24 h post exercise vs. pre-exercise for the normalized expression levels (significance analysis of microarrays, SAM). Fold changes are given in color coding: Red: up, blue: down. Arrows indicates the transcripts, which are associated with angiogenesis.

We explored the expression of 231 selected transcripts post exercise in a subset of 12 participants for an influence of SNP rs2104772. The expression levels of 124 gene transcripts, including *tenascin-C*, were affected over the course of the first 24 h into recovery from the first bout of exercise (S1 Fig). Twenty-four hours after exercise, when the next exercise bout would normally apply during training, abundances of 34 gene transcripts showed SNP rs2104772 dependent alterations 24 hours post exercise (Fig 6). Eight transcripts being associated with angiogenesis were oppositely expressed between the A/A and T/T genotype. Four of which (Ang 1, VEGF A, VEGF-R2 and VIM) were increased in the A/A genotype 24 hours after endurance exercise and reducibly expressed, or unaltered, in the T/T genotype (Table 3).

**Table 3:** Transcripts with an rs2104772 related expression response to exercise.

Gene name	Genbank	Gene ontology	Gene ontology	Gene ontology	T/T	A/T	A/A
	biological	biological process	cellular location				
Ang 1	U83508	angiogenesis	adhesion	extracelllular	0.89	1.52	2.49
EPAS1	U81984	angiogenesis	transcription	nucleus	1.34	1.07	0.97
FGF R4	L03840	angiogenesis	cell migration	plasma membrane	2.69	0.87	0.61
PGF	X54936	angiogenesis	Signaling	extracellular	1.65	1.13	0.80
TIMP1	X03124	angiogenesis	protein turnover	extracellular	2.32	0.97	0.82
VEGF A	M32977	angiogenesis	Signaling	extracellular	0.97	1.29	1.13
VEGF-R2	AF063658	angiogenesis	Signaling	plasma membrane	0.91	1.29	1.54
VIM	X56134	angiogenesis	cytoskeletal protein	cytoplasm	1.02	1.76	2.90
ACAT1	M74096	metabolism	mitochondrial metabolism	mitochondria	1.01	1.21	1.29
CA3	M29458	metabolism	mitochondrial metabolism	cytoplasm	0.77	1.10	1.13
CPTI	D87812	metabolism	mitochondrial metabolism	mitochondria	1.71	1.07	0.92
LCAD	M74096	metabolism	mitochondrial metabolism	mitochondria	1.88	1.16	1.01
LPL	M15856	metabolism	mitochondrial metabolism	extracellular	1.73	1.19	1.01
MB	M14603	metabolism	mitochondrial metabolism	sarcoplasm	0.72	1.00	1.04
PDHA2	M86808	metabolism	mitochondrial metabolism	mitochondria	1.69	0.88	1.01
UCP-3	AF011449	metabolism	mitochondrial metabolism	membrane	1.21	0.59	0.46
FATP	AF055899	metabolism	fatty acid metabolism	sarcoplasm	0.82	1.29	1.42
ALDO C	AF054987	metabolism	carbohydrate metabolism	sarcoplasm	1.63	1.01	0.90
PGK1	V00572	metabolism	carbohydrate metabolism	cytoplasm	0.85	1.09	1.22
GST-3	AF026977	metabolism	detoxification	intracellular	0.86	1.06	1.18
SOD1	M13267	metabolism	radical metabolism	sarcoplasm	1.02	1.44	1.60
SOD2	M36693	metabolism	radical metabolism	mitochondria	1.04	1.24	1.35
ACTN2	M86406	myogenesis	sarcomere assembly	cytoskeleton	0.96	1.16	1.42
28S rRNA	M11167	protein turnover	protein synthesis	cytoplasm	1.16	0.76	0.99
CCT1	X52882	protein turnover	protein folding	sarcoplasm	1.93	1.03	0.92
RPS29	U14973	protein turnover	protein synthesis	cytoplasm	0.58	1.09	1.16
RPS9	U14971	protein turnover	protein synthesis	cytoplasm	0.83	1.31	1.70
ubiquitin C	M26880	protein turnover	protein synthesis	cytoplasm	0.64	1.33	1.34
c-jun	J04111	regulation	signal transduction	nucleus	0.95	1.11	1.33
CCNA1	U66838	regulation	cell cycle	nucleus	1.57	0.97	0.94
HIF-1b	M69238	regulation	transcription	nucleus	1.50	1.06	1.04
Il-6	X04602	regulation	Signaling	extracellular	2.03	1.05	0.93
p19	U40343	regulation	cell cycle	nucleus	2.15	1.07	1.01
PPARA	L02932	regulation	transcription	nucleus	1.63	0.93	0.84

Gene name, Genbank identifiers and gene ontologies for the 34 transcripts, which demonstrated different expressional regulation 24 h post exercise between rs2104772 genotypes. Values represent mean fold changes 24 h post exercise vs. pre-exercise levels.

### Vimentin and VEGF A protein in relation to SNP rs2104772

**Fig 7:**
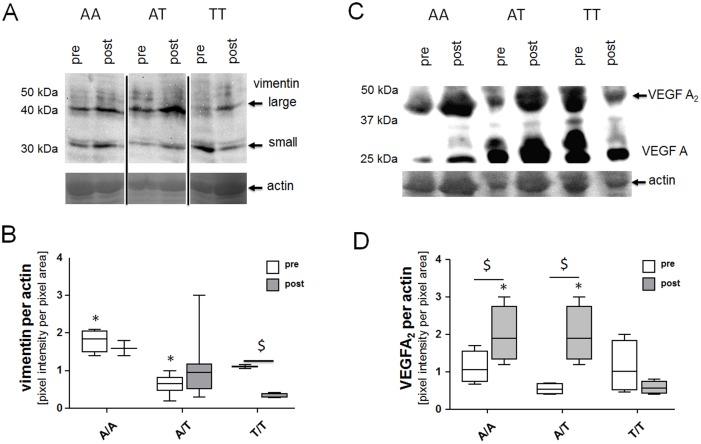
Vimentin and VEGF A protein in vastus lateralis muscle with endurance training. (*A*, *B*) Immunoblot showing the small (32 kDa) and large (45 kDa) vimentin isoforms (A) and VEGF A monomers and dimer (B) in one participant for each genotype before and after endurance training. The position of the actin band on the Ponceau-S-stained membrane before immunoblotting, which served as a loading control, is indicated. For image assembly see S5, S6, S7, S8, S9 and S10 Figs. (C, D) Box whisker plot visualizing the median ± standard error (central lines and boxes, respectively) and minima/maxima (whiskers) of the fold changes in vimentin (C) and VEGF A content (D). In total biopsies from 18 subjects were assessed: A/A (*n* = 4), A/T (*n* = 10), and T/T (*n* = 4). *, *p* < 0.05 vs. T/T same time point; $, p < 0.05 vs. pre. Repeated ANOVA with Fisher’s post-hoc test.

We assessed alterations in VIM (vimentin) at the protein level, because the response of its transcript to exercise was strongest (i.e. 2.9-fold) affected by SNP rs2104772 between the A/A and T/T genotype. A trend for an interaction effect between genotype and training was evident for the total content of vimentin protein (p = 0.06). Vimentin protein was 1.7-fold more abundant in the A/A than the T/T genotype before training (Fig 7). Vimentin content was 3.1-fold reduced in T/T homozygotes after endurance training but unaffected by endurance training in A/A homozygotes and A/T heterozygotes. Likewise regulation of the protein content of the dimer of the pro-angiogenic factor VEGF A showed genotype dependent regulation (p = 0.01). The protein content of the VEGF A dimer was increased in carriers of the T-allele, only, after endurance training.

## Discussion

In the current study, we tested whether endurance training increases tenascin-C expression in association with structural adjustments in muscle and whether this response would be modified by SNP rs2104772, which was predicted to affect adhesive function and the molecular interactions of tenascin-C protein [31, 32]. We provide first evidence that the content of tenascin-C in knee extensor muscle of adult men is increased after repeated bouts of a concentric type of endurance exercise, which is not associated with muscle damage [27, 41]. We find that within the studied subjects the T/T genotype of SNP rs2140772 was less effective in increasing capillary-to-fiber ratio than A/A and A/T genotypes after the 6 weeks of training. This result coincided with a) enhanced protein content of tenascin-C in association with venules and arterioles, b) rs2140772-related changes of 34 gene transcripts including 8 being associated with angiogenesis 24 h after an acute bout of exercise, and c) lower *tenascin-C* transcript and protein abundances at baseline and a blunted effect of training on tenascin-C protein content in T/T homozygotes, d) a specific reduction in the protein content of vimentin and the VEGF A dimer in T/T homozygotes after the endurance training program. In line with our hypothesis, our data provide evidence that A/A homozygosity of rs2104772 positively influences improvements in muscle capillarization achieved with endurance exercise. Our observations indicate that blood vessel related alterations in the expression of tenascin-C is a specific response to cycling type endurance training which may vary between genotypes of rs2104772 and relates to variability in adjustments of capillarization with endurance training.

The present novel findings also identify an effect of SNP rs2104772 on the volume density of subsarcolemmal and myofibrillar mitochondria prior to training (Table 1). Notwithstanding, the training response did not include genotype dependent alterations in mitochondrial volume density (Table 2). Microarray analysis did however identify SNP rs2104772-modulated expression of mitochondria related gene transcripts in the 24-h response of *vastus lateralis* muscle to endurance exercise in the investigated subset of subjects (Table 3). This molecular observation is consistent with the reported connection between adjustments in the volume density of mitochondria and capillaries with training [21]. The observed absence of genotype modulated training effects on mitochondria—over the entire study population of 61 subjects—indicates that additional, unidentified human factors confound a possibly rs2104772-related expressional regulation of mitochondria by endurance-training. In this regard it is of note that the inheritance of the mitochondrial genome deviates from a Hardy-Weinberg equilibrium [45], suggesting that the identified trend (i.e. p = 0.059) for a rejection of a Hardy-Weinberg equilibrium for SNP rs2104772 may reflect this influence.

The T-to-A exchange in the *tenascin-C* gene due to s2104772 leads to the exchange of leucine by isoleucine in the 13^th^ fibronectin type III domain of tenascin-C [31], which interferes with fibrinogenase’s and wound healing [46]. Based on structural modelling it was predicted that the presence of isoleucine rather than leucine causes beta-sheet instability and may negatively affect tenascin-C molecular elasticity, and potentially reduce the capacity of tenascin-C to dissolve cell—matrix contacts [31, 32]. The presently identified effects of SNP rs2104772 on muscle capillarization are a further indication for the functional relevance of the resultant amino acid exchange. Nevertheless, experimental proof for this molecular scenario needs to be investigated. In this regard the lowered *tenascin-C* transcript and protein abundances at baseline in the T/T homozygotes suggest an influence of rs2104772 on the expression and/or stability of tenascin-C itself. In line with previous studies (reviewed in [33]), reporting *tenascin-C* genotype-dependent remodeling of the airway endothelium in asthma, the A/A genotype showed superior gains in muscle capillarization post-exercise as compared to the T/T genotype. Hence, the observed larger increase in capillary-to-fiber ratio in the A/A and A/T genotype compared to the T/T genotype of rs2107772 call for future studies to explore the mechanistic foundation.

Changes in the capillary-to-fiber ratio are understood to reflect effective capillary remodeling [19]. Thus, the effects of SNP rs2104772 on gains in capillary-to-fiber ratio with endurance training (Fig 3) support that tenascin-C is actively and specifically involved in the processes that lead to exercise-induced capillary growth. By contrast, structural parameters related to mitochondria and myofibrils were not affected in a genotype-specific way by endurance training (Table 2). The observed effect of SNP rs2104772 was related to a lower content of the tenascin-C protein in T/T genotype compared to the A/T and A/A genotype before and after endurance training. Together with the detected association of tenascin-C expression with blood vessels, and the comparable capillarization in genotypes prior to training, the findings imply that blood vessels show permanent differences in the content of this anti-adhesive protein. This lends further credence to the notion that the function of tenascin-C is associated with the activity-induced structural rearrangement of capillaries with respect to muscle fibers.

Microarray-based analysis of transcript expression pointed out 34 gene transcripts, which expression after a bout of endurance exercise was specifically modulated by SNP rs2104772 (Table 3). The detailed inspection revealed that 8 of these were associated with angiogenesis; thus indicating that the reduced gains in capillary-to-fiber ratio in extensor muscle of the T/T genotype after endurance training are reflected by a selective altered angiogenic response during recovery single exercise.

The rs2104772 dependent expression of angiogenesis associated transcripts is of interest in comparison to their expressional regulation in endothelial and smooth muscle cells in response to shear stress. For instance the increase in transcript levels of vascular endothelial growth factor A (VEGF A), its receptor (VEGF-R2), and VIM in the A/A genotype 24 after exercise is reminiscent to the reported up-regulation of these transcripts in endothelial and smooth muscle cells by shear stress (compare Table 3 with [47–51]. Correspondingly, the reduced transcript level response of the angiogenesis-associated transcription factors EPAS1 and FGF- R4 in the A/A genotype compares to the down-regulation of this transcript levels by shear stress [52] and the down-regulated FGF R4 phosphorylation by shear stress [53]. Therefore one interpretation of our findings may be that the rs2104772-dependent response of angiogenesis-related transcript expression post-exercise in *m*. *vastus lateralis* reflects the perfusion related effect of contraction on capillary remodeling [54].

It is worth noting that we examined tenascin-C-associated angiogenesis in the health-associated situation of exercise. This observation differs from those in studies assessing the role of tenascin-C in vascular growth in pathological blood vessel remodeling in chronic angiotensin-mediated hypertension and tumor growth [4, 5, 7] which are characterized by chronic inflammation and elevated microvascular permeability [55, 56]. The latter has been shown to reflect leaky blood vessels in association with the angiopoietin pathway [57]. Intriguingly the increased expression of transcripts for angiopoietin (i.e. Ang 1), VEGF A and VIM, in exercised muscle of the A/A and A/T genotype relates to similar regulation in other tissues in the condition of elevated blood pressure. For instance, there is an increased abundance of Ang 1 and VEGF A proteins in blood serum of the hypertensive patient [58]. Interestingly as well, vimentin content in endothelial cells correlates with hemodynamic parameters [59] and a secreted form of the vimentin protein specifically localizes to the endothelial surface of blood capillaries and small veins [60]. Certainly, further investigations are required to explain the observed similarity in alterations of the related factors in the studied tissues (i.e. blood serum, endothelium and skeletal muscle) between the hypertensive patient and the exercised subject and their relevance for the transport of blood-borne substrates. In this regard, the identified relationship between the selective reduction in vimentin and VEGF A content in T/T homozygotes (Fig 7) and the unaffected transcript expression post exercise is of interest (Table 3, Fig 6). This regulation differed to the increase in corresponding VIM transcript post exercise in A/T and A/A genotypes, which maintained vimentin and VEGF A content after the 6 weeks of training. Accordingly, the repeated increase in vimentin transcript expression post exercise in A-allele carriers would be compatible with an enhanced capacity for the synthesis of vimentin and VEGF A protein, which would allow maintaining protein levels of the respective proteins after training in contrast to T-homozygotes.

Obviously, certain limitations apply when drawing mechanistic conclusions from a sports-medical intervention in humans that involves comparative genetic analyses but which does not introduce genetic alterations *de novo*. Additionally, the number of participants included in our study was low compared to many genetic studies, thus setting possible limitations for the representativeness of the observed genotype effect for the general population. This restriction was related to the invasive nature of the study—requiring an otherwise unnecessary collection of muscle biopsies—ethically bound to use as few subjects as possible. Additionally, small effects sizes for alterations in the integrated variables of maximal oxygen uptake or maximal aerobic power output compared to alterations in aerobic metabolism at the muscle level [21], may explain why the identified genotype differences in transcript expression and capillarization did not manifest in functional adjustments at the system level. Our human investigation is underpowered to demonstrate a causal implication of rs2104772 in moderating angiogenesis because, for obvious reasons, we did not manipulate the A/T nucleotides within SNP rs2104772. Nevertheless, a post hoc analysis indicates that the power values for the observed rs2104772 genotype-specific alterations in capillary-to-fiber ratio (0.93) and the alterations in protein content of the pro-angiogenic factors VIM (0.98) and VEGF A (0.93) after training are reasonably high (S1 Table). These statistical results qualify the rs2104772-specific transcript alterations post exercise, as suggested using explorative transcript profiling, and the rs2104772-specific adjustments in muscle capillarization after training, and are consistent with the suggested implication of tenascin-C in capillary remodeling in humans.

## Conclusions

Our results confirm the suggested association of tenascin-C and the non-synonymous T-to-A exchange in amino acid codon 1677 within the *tenascin-C* gene due to SNP rs2104772 with exercise-induced capillary growth. Further studies are required to explore the relevance of the identified association of rs2104772 with angiogenesis in exercised muscle beyond the studied group of subjects. Equally the biological pathway underlying the rs2104772-related dependence of angiogenic transcript expression after concentric type endurance exercise and increased capillary-to-fiber ratio achieved with repeated exercise, as well as the implications for local alterations in the muscle microvasculature with physical training remains to be addressed. Collectively our results highlight that a genetical approach, which characterizes muscle plasticity to exercise using classical morphometry and transcript profiling, is suitable to explore mechanistically important molecular regulation in humans.

## Supporting information

S1 Fig Transcript response to single exercise. Line graph of mean alterations of the 124 gene transcripts which were affected during the course of the first 24 hours of recovery from a single bout of endurance exercise as revealed with a one class time course (SAM) at a FDR of 1%. Transcripts and their Genbank number are grouped according to their response pattern.
(TIF)

S2 Fig Uncropped actin blot Fig 4A. Original image showing the Ponceau S stained membrane after western blotting of 6 separated pre/post sample pairs. Molecular weight markers were loaded to the left and right.
(TIF)

S3 Fig Uncropped tenascin-C blot Fig 4A. Original image showing the detetection of tenascin-C in on the western blotted membrane with 6 pre/post sample pairs. The molecular weight markers to the right was trimmed off before detection.
(TIF)

S4 Fig Image assembly of Fig 4A. Image assembly of the tenascin-C-stained and Ponceau S stained membrane with the 6 pre/post sample pairs, respectively, with the explanation of the applied labelling and cropping.
(TIF)

S5 Fig Uncropped actin blot Fig 7A. Original image showing the Ponceau S stained membrane after western blotting of 4 separated pre/post sample pairs. Molecular weight marker was loaded to the left.
(TIF)

S6 Fig Uncropped vimentin blot Fig 7A. Original image showing the detetection of vimentin on the western blotted membrane with 4 pre/post sample pairs. Molecular weight marker was loaded to the left.
(TIF)

S7 Fig Image assembly Fig 7A. Image assembly of the vimentin-stained and Ponceau S stained membrane with the 4 pre/post sample pairs, respectively, with the explanation of the applied labelling and cropping.
(TIF)

S8 Fig Uncropped actin blot Fig 7B. Original image showing the Ponceau S stained membrane after western blotting of 6 separated pre/post sample pairs. Molecular weight markers were loaded to the left and right.
(TIF)

S9 Fig Uncropped VEGFA blot Fig 7B. Original image showing the detetection of VEGFA on the western blotted membrane with 6 pre/post sample pairs. Molecular weight markers were loaded to the left and right.
(TIF)

S10 Fig Image assembly Fig 7B. Original image showing the detetection of VEGFA on the western blotted membrane with 6 pre/post sample pairs. Molecular weight marker was loaded to the left.
(TIF)

S1 Table Post hoc power analysis. Effect size and power of muscle parameters, demonstrating a genotype effect on exercise/training induced alterations, as estimated by post hoc power analysis using G*power. The analysis was carried out with the following settings: Test family, F tests; statistical test, ANOVA: repeated measures, between factors; type of power analysis, Post hoc. Values were imputed using the mean of values pre and post exercise or training, respectively, the standard deviation over all values and correlations between pre and post samples.
(DOCX)
