# Supplementary figures and images for: T/T homozygosity of the tenascin-C gene polymorphism rs2104772 negatively influences exercise-induced angiogenesis
Source: PLoS One. 2017 Apr 6;12(4):e0174864. doi: 10.1371/journal.pone.0174864 (PMC5383042; doi:10.1371/journal.pone.0174864)

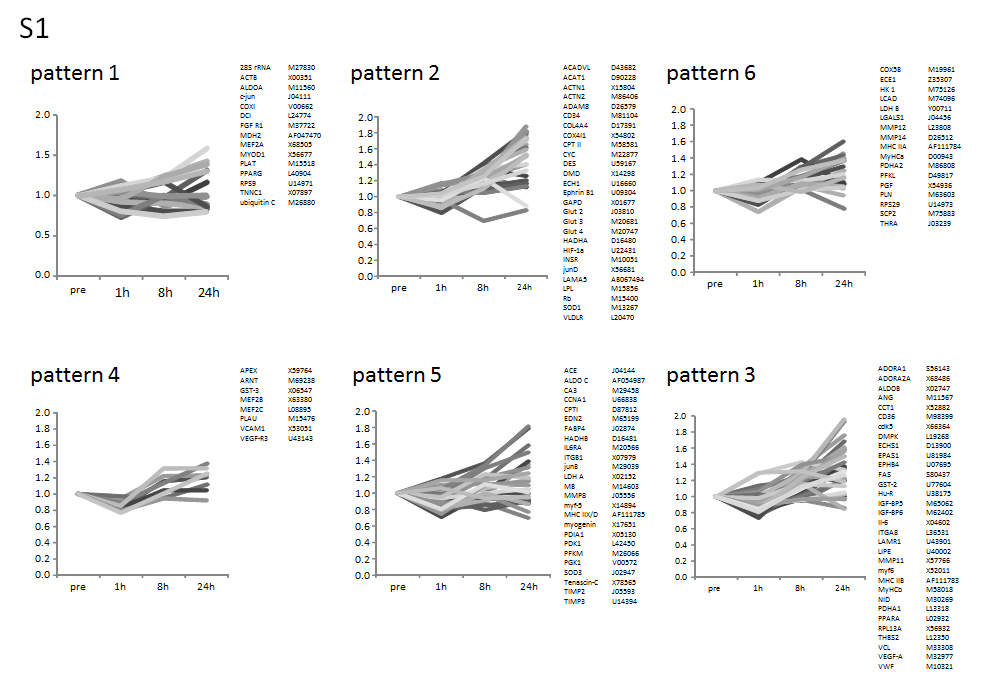

Supplement: S1 Fig — Line graph of mean alterations of the 124 gene transcripts which were affected during the course of the first 24 hours of recovery from a single bout of endurance exercise as revealed with a one class time course (SAM) at a FDR of 1%. Transcripts and their Genbank number are grouped according to their response pattern. (TIF) [file pone.0174864.s001.tif]

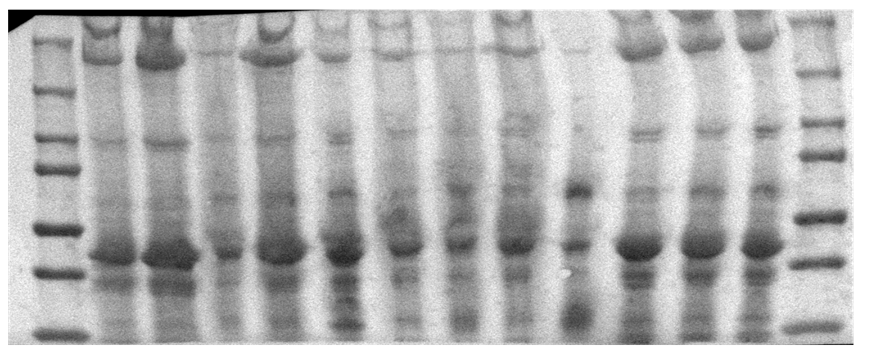

Supplement: S2 Fig — Original image showing the Ponceau S stained membrane after western blotting of 6 separated pre/post sample pairs. Molecular weight markers were loaded to the left and right. (TIF) [file pone.0174864.s002.tif]

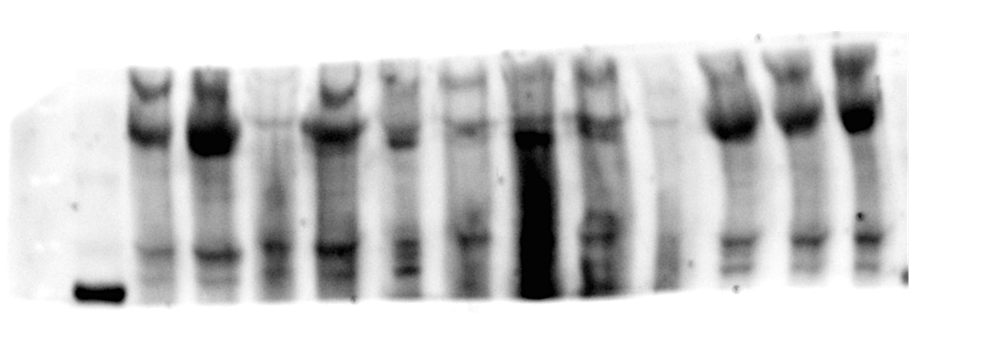

Supplement: S3 Fig — Original image showing the detetection of tenascin-C in on the western blotted membrane with 6 pre/post sample pairs. The molecular weight markers to the right was trimmed off before detection. (TIF) [file pone.0174864.s003.tif]

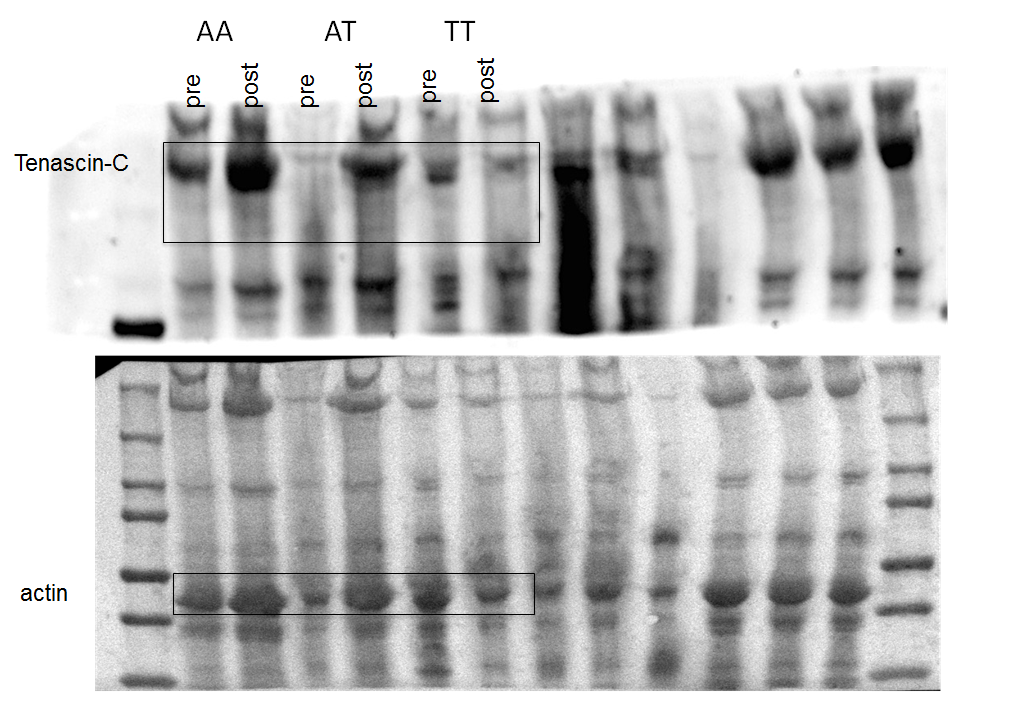

Supplement: S4 Fig — Image assembly of the tenascin-C-stained and Ponceau S stained membrane with the 6 pre/post sample pairs, respectively, with the explanation of the applied labelling and cropping. (TIF) [file pone.0174864.s004.tif]

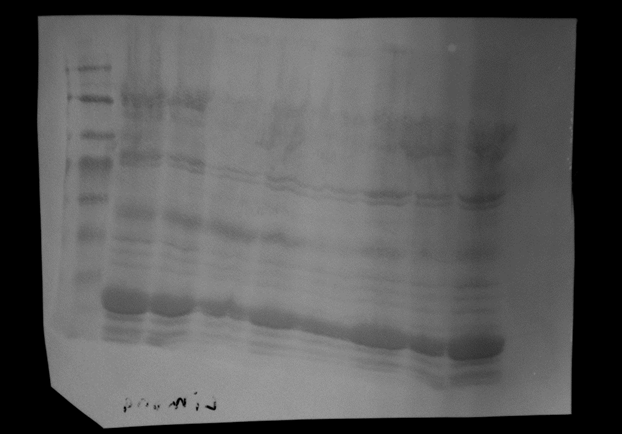

Supplement: S5 Fig — Original image showing the Ponceau S stained membrane after western blotting of 4 separated pre/post sample pairs. Molecular weight marker was loaded to the left. (TIF) [file pone.0174864.s005.tif]

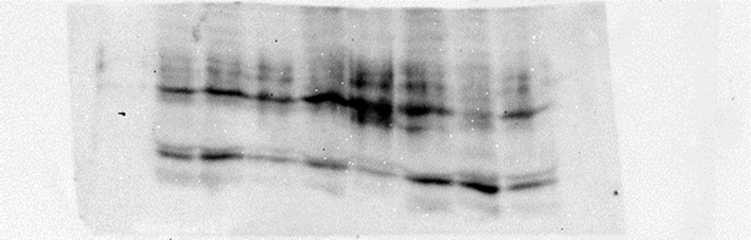

Supplement: S6 Fig — Original image showing the detetection of vimentin on the western blotted membrane with 4 pre/post sample pairs. Molecular weight marker was loaded to the left. (TIF) [file pone.0174864.s006.tif]

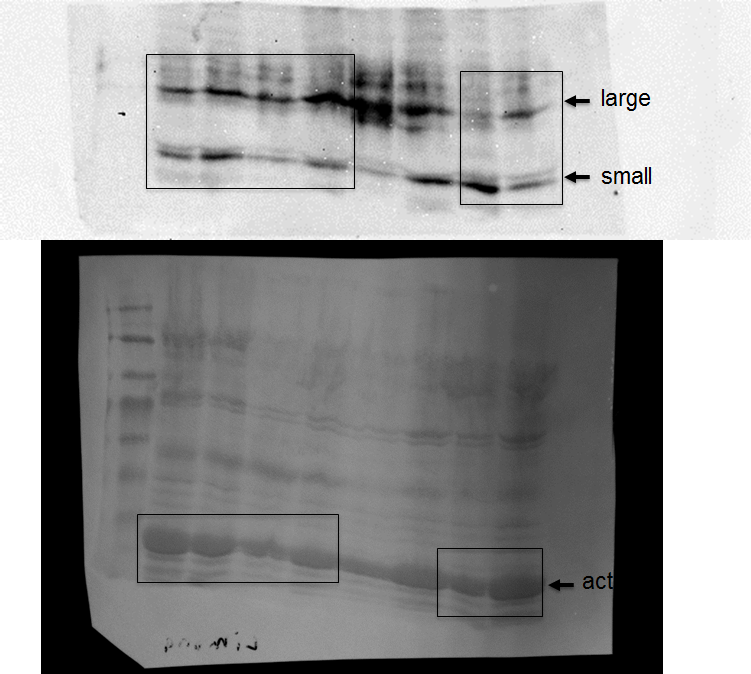

Supplement: S7 Fig — Image assembly of the vimentin-stained and Ponceau S stained membrane with the 4 pre/post sample pairs, respectively, with the explanation of the applied labelling and cropping. (TIF) [file pone.0174864.s007.tif]

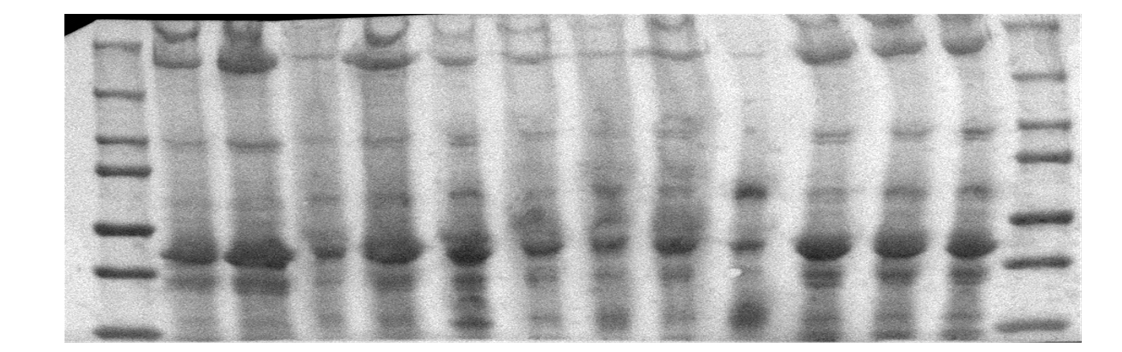

Supplement: S8 Fig — Original image showing the Ponceau S stained membrane after western blotting of 6 separated pre/post sample pairs. Molecular weight markers were loaded to the left and right. (TIF) [file pone.0174864.s008.tif]

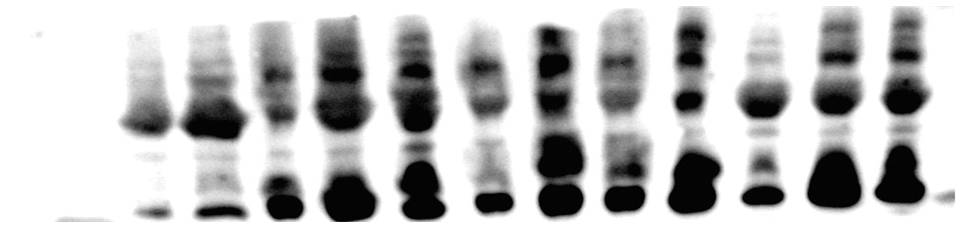

Supplement: S9 Fig — Original image showing the detetection of VEGFA on the western blotted membrane with 6 pre/post sample pairs. Molecular weight markers were loaded to the left and right. (TIF) [file pone.0174864.s009.tif]

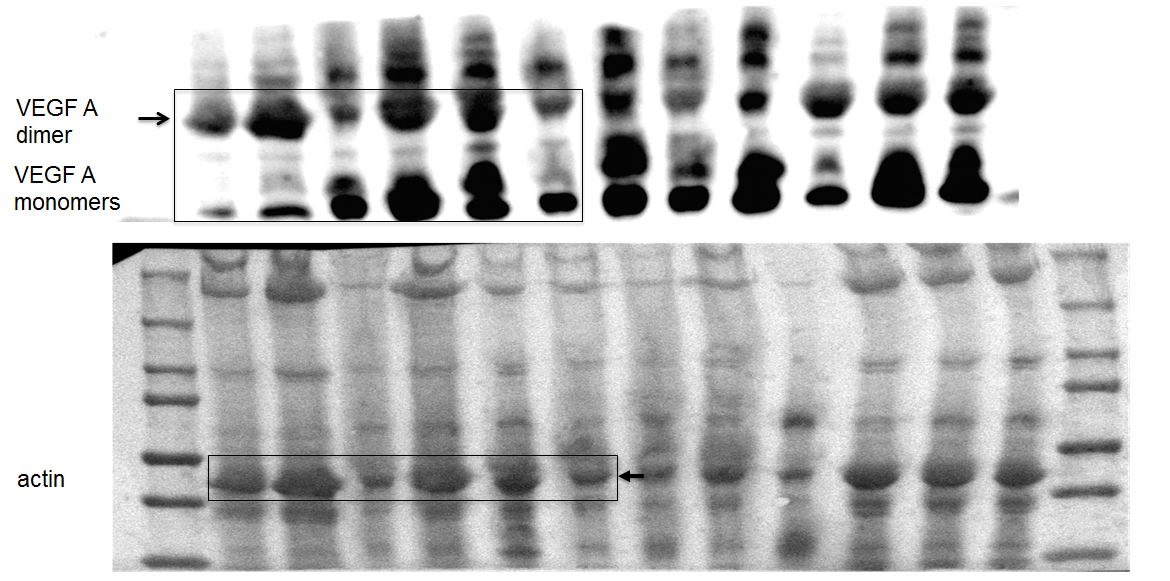

Supplement: S10 Fig — Original image showing the detetection of VEGFA on the western blotted membrane with 6 pre/post sample pairs. Molecular weight marker was loaded to the left. (TIF) [file pone.0174864.s010.tif]
