# Supplementary material for: T/T homozygosity of the tenascin-C gene polymorphism rs2104772 negatively influences exercise-induced angiogenesis
Source: PLoS One. 2017 Apr 6;12(4):e0174864. doi: 10.1371/journal.pone.0174864 (PMC5383042; doi:10.1371/journal.pone.0174864)
Supplement: S1 Table — Effect size and power of muscle parameters, demonstrating a genotype effect on exercise/training induced alterations, as estimated by post hoc power analysis using G*power. The analysis was carried out with the following settings: Test family, F tests; statistical test, ANOVA: repeated measures, between factors; type of power analysis, Post hoc. Values were imputed using the mean of values pre and post exercise or training, respectively, the standard deviation over all values and correlations between pre and post samples. (DOCX) [file pone.0174864.s011.docx]

**Supplemental table 1: Post hoc power analysis**

Effect size and power of muscle parameters, demonstrating a genotype effect on exercise/training induced alterations, as estimated by post hoc power analysis using G*power. The analysis was carried out with the following settings: Test family, F tests; statistical test, ANOVA: repeated measures, between factors; type of power analysis, Post hoc. Values were imputed using the mean of values pre and post exercise or training, respectively, the standard deviation over all values and correlations between pre and post samples.

| ***factor*** | ***class*** | ***interaction effect of rs2104772 with*** | ***effect size*** | ***power*** |
| --- | --- | --- | --- | --- |
| capillary-to-fiber ratio | *cellular* | *training response* | 0.69 | 0.98 |
|  |  |  |  |  |
| VIM | *protein* | *training response* | 0.68 | 0.93 |
| VEGF A | *protein* | *training response* | 0.80 | 0.98 |
| Tenascin C | *protein* | *training response* | 0.47 | 0.93 |
|  |  |  |  |  |
| VIM | *transcript* | *exercise response* | 0.86 | 0.91 |
| VEGF A | *transcript* | *exercise response* | 0.60 | 0.49 |
| Tenascin C | *transcript* | *exercise response* | 0.68 | 0.62 |
